# Guest Editorial Introduction to the Special Section on Weakly-Supervised Deep Learning and Its Applications

**DOI:** 10.1109/OJEMB.2024.3404653

**Published:** 2024-05-23

**Authors:** Yu-Dong Zhang

**Affiliations:** King Abdulaziz University 37848 Jeddah Saudi Arabia

## Abstract

Researchers in biomedical engineering are increasingly turning to weakly-supervised deep learning (WSDL) techniques [1] to tackle challenges in biomedical data analysis, which often involves noisy, limited, or imprecise expert annotations [2]. WSDL methods have emerged as a solution to alleviate the manual annotation burden for structured biomedical data like signals, images, and videos [3] while enabling deep neural network models to learn from larger-scale datasets at a reduced annotation cost. With the proliferation of advanced deep learning techniques such as generative adversarial networks (GANs), graph neural networks (GNNs) [4], vision transformers (ViTs) [5], and deep reinforcement learning (DRL) models [6], research endeavors are focused on solving WSDL problems and applying these techniques to various biomedical analysis tasks.

Researchers in biomedical engineering are increasingly turning to weakly-supervised deep learning (WSDL) techniques [1] to tackle challenges in biomedical data analysis, which often involves noisy, limited, or imprecise expert annotations [2]. WSDL methods have emerged as a solution to alleviate the manual annotation burden for structured biomedical data like signals, images, and videos [3] while enabling deep neural network models to learn from larger-scale datasets at a reduced annotation cost. With the proliferation of advanced deep learning techniques such as generative adversarial networks (GANs), graph neural networks (GNNs) [4], vision transformers (ViTs) [5], and deep reinforcement learning (DRL) models [6], research endeavors are focused on solving WSDL problems and applying these techniques to various biomedical analysis tasks.

The special section of the IEEE Open Journal of Engineering in Medicine and Biology, entitled "Weakly-Supervised Deep Learning and its Applications," is devoted to exploring and highlighting the latest advancements in WSDL techniques, specifically within the context of biomedical engineering and medicine. This special section seeks to address the challenges inherent in biomedical data analysis, which often involves noisy, limited, or imprecise annotations. By focusing on WSDL methods, the special section aims to showcase innovative WSDL approaches that can effectively learn from large-scale biomedical data while minimizing the need for extensive manual annotation efforts. The ultimate goal is to advance the field of biomedical engineering by promoting the development and application of WSDL techniques.

The objective of the study from Liu, et al. [A1] is to enhance a small and imbalanced wound dataset by employing semi-supervised learning techniques with the assistance of a secondary dataset. Subsequently, the augmented wound dataset is utilized for deep learning-based wound assessment. The Photographic Wound Assessment Tool (PWAT), a clinically validated method, is employed to evaluate eight attributes of wounds comprehensively. These attributes include Size, Depth, Necrotic Tissue Type, Necrotic Tissue Amount, Granulation Tissue Type, Granulation Tissue Amount, Edges, and Periulcer Skin Viability. The study utilizes a reference corpus comprising 1639 labeled wound images with ground truth PWAT scores. Semi-supervised learning techniques and a Progressive Multi-Granularity training mechanism are applied to leverage a secondary dataset comprising 9870 unlabeled wound images. The wound scoring process utilizes the EfficientNet Convolutional Neural Network on the augmented wound corpus. The results indicate that the proposed Semi-Supervised PMG EfficientNet (SS-PMG-EfficientNet) approach achieves classification accuracies and F1 scores of approximately 90% on average for all eight PWAT sub-scores. Furthermore, the SS-PMG-EfficientNet approach outperforms a range of baseline models and demonstrates a 7% improvement over the prior state-of-the-art method (without data augmentation).

Emotion recognition has emerged as a crucial task in human-machine interaction, garnering increasing attention over the years. Pan, et al. [A2] present Deep-Emotion, a novel deep learning-based multimodal emotion recognition (MER) framework designed to dynamically integrate discriminative features from facial expressions, speech, and electroencephalogram (EEG) signals to enhance MER performance. Deep-Emotion comprises three branches: the facial, speech, and EEG branches. The facial branch utilizes an enhanced GhostNet neural network for feature extraction, mitigating overfitting during training and improving classification accuracy compared to the original GhostNet network. The speech branch employs a lightweight, fully convolutional neural network (LFCNN) for efficient speech emotion feature extraction. For the EEG branch, a tree-like LSTM (tLSTM) model is proposed to fuse multi-stage features for EEG emotion feature extraction. Decision-level fusion is employed to integrate the recognition outcomes from the three modalities, resulting in more comprehensive and accurate performance. Extensive experiments conducted on the CK+, EMO-DB, and MAHNOB-HCI datasets validate the efficacy of the Deep-Emotion approach, demonstrating its superior performance and the feasibility of the MER methodology.

The aim of Karthik, et al. [A3] is to explore the utility of uncertainty estimations derived from approximate Bayesian inference in elucidating the behavior of deep neural networks. Particularly in unsupervised learning scenarios lacking expert annotations, the assessment of model performance via uncertainties becomes increasingly critical. To address this, the authors propose a proof-of-concept approach for extending the estimation of aleatoric and epistemic uncertainties in unsupervised magnetic resonance (MR) to computed tomography (CT) synthesis for scoliotic spines. The authors introduce a novel adaptation of the cycle-consistency constraint within the CycleGAN framework, enabling the prediction of aleatoric uncertainty maps alongside the conventional volume-to-volume translation between MR and CT data. The study conducted ablation experiments to investigate the role of uncertainty estimation as an implicit regularizer and a metric of the model's confidence.

Langarica, et al. [A4] utilize physiological data gathered from wearable sensors to construct a series of data-driven models employing deep learning methodologies. A systematic comparative analysis of these models is undertaken to provide valuable insights for practitioners and researchers engaging in glucose prediction using deep learning techniques. Key inquiries addressed in this investigation encompass the evaluation of diverse deep learning architectures, identification of the optimal set of input variables for precise glucose prediction, comparison among population-wide, fine-tuned, and personalized models, and examination of the impact of individual data volume on model performance. Furthermore, the study introduces a meticulously curated dataset comprising data from both healthy individuals and those with diabetes, collected under free-living conditions. This dataset aims to promote research in this domain and facilitate equitable comparisons among researchers.

In this study by Jhang, et al. [A5], the authors introduce a novel gastric section correlation network (GSCNet) designed for the Computer-Aided Gastric Intestinal metaplasia (CGI) diagnosis using endoscopic images of three principal gastric sections: antrum, body, and cardia. GSCNet comprises two primary modules: the scaling feature fusion module and the section correlation module. The former is dedicated to extracting scaling fusion features and is adept at effectively representing mucosal characteristics under varying viewing angles and scale modifications for each gastric section. Meanwhile, the latter module leverages medical prior knowledge, incorporating three-section correlation losses to model the interdependencies among different gastric sections for CGI diagnosis. Their experimental results demonstrate that the proposed method outperforms existing deep learning approaches, achieving notably high testing accuracy, sensitivity, and specificity metrics of 0.957, 0.938, and 0.962, respectively.

Expanding upon the authors' prior research, Toubal, et al. [A6] introduce EDNet, a novel deep learning-based approach for cell detection, tracking, and motility analysis. EDNet exhibits enhanced robustness to cellular morphology variations across diverse cell lines while effectively modeling cell lineage and proliferation dynamics. Leveraging an ensemble methodology for 2D cell detection, EDNet achieves superior performance compared to single-model YOLO and FasterRCNN convolutional neural networks, demonstrating state-of-the-art results. The detected cells by EDNet are subsequently utilized in their M2Track multiobject tracking algorithm, facilitating the tracking of cells, identification of cell mitosis events, and generation of cell lineage graphs. Their methodologies exhibit state-of-the-art performance on the Cell Tracking and Mitosis (CTMCv1) dataset, achieving a Multiple Object Tracking Accuracy (MOTA) score of 50.6% and a tracking lineage graph edit (TRA) score of 52.5%. Furthermore, the authors conduct comparative analyses between our detection and tracking methods and human performance on external datasets, particularly focusing on the motility of muscle stem cells under various physiological and molecular stimuli.

Liu, et al. [A7] aim to explore the utility of graph attention networks in the recognition of autism spectrum disorders (ASD). To achieve this, the authors propose a novel approach called the node features graph attention network (NF-GAT), which is designed to learn functional connectivity (FC) features for ASD diagnosis. Initially, node features are derived from functional magnetic resonance imaging (fMRI) data, wherein each subject is represented as a graph. Subsequently, the graph attention layer is employed to extract informative node features, facilitating ASD classification based on the node information from different nodes. Their experimental results demonstrate that the NF-GAT outperforms other existing models, exhibiting significant advantages in terms of classification accuracy. In conclusion, the NF-GAT framework proves to be an effective tool for ASD classification.

Ren, et al. [A8] present a novel underlying knowledge-based semi-supervised learning framework termed UKSSL, comprising two integral components: MedCLR, which extracts feature representations from unlabeled datasets, and UKMLP, which utilizes these representations and refines them using limited labeled data for medical image classification. UKSSL is evaluated on the LC25000 and BCCD datasets, utilizing only 50% labeled data. Notably, it achieves precision, recall, F1-score, and accuracy metrics of 98.9% and 94.3%, 94.5%, 94.3%, and 94.1% on LC25000 and BCCD, respectively. These performance metrics surpass those attained by other supervised learning methods operating with 100% labeled data. In conclusion, UKSSL demonstrates proficiency in extracting underlying knowledge from unlabeled datasets and exhibits superior performance when utilizing limited labeled medical images.

The special section encompasses a range of innovative research endeavors in machine learning applied to medical domains. Addressing diverse challenges, these authors explore techniques such as semi-supervised learning for dataset enhancement, multimodal emotion recognition integrating facial expressions, speech, and EEG signals, uncertainty estimation in deep neural networks, glucose level prediction using wearable sensor data, and advanced methodologies for medical image analysis including wound assessment, disease diagnosis, and cell detection and tracking. These investigations collectively highlight the potential of machine learning approaches [7] to revolutionize healthcare [8] by improving diagnostic accuracy, treatment monitoring [9], and understanding complex medical conditions.

We extend our sincere gratitude to the researchers who have contributed to this special section. We highly appreciate the assistance provided by the editorial assistants in managing these papers, and we wish to express our gratitude to all anonymous reviewers for their candid assessments, insightful comments, and beneficial recommendations. We also extend our gratitude to the Editor-in-Chief for his guidance and support throughout the publication process.

## Conflict of Interest

The author declares that he has no conflicts of interest to report regarding this editorial.

## Author Contribution

Y.D. Zhang wrote this editorial.


Yu-Dong Zhang, 
Guest Editor
King Abdulaziz University
Jeddah, Saudi Arabia
yudongzhang@ieee.org


## Appendix Related Work

[A1] Z. Liu, E. Agu, P. Pedersen, C. Lindsay, B. Tulu, and D. Strong, “Chronic wound image augmentation and assessment using semi-supervised progressive multi-granularity EfficientNet,” *IEEE Open J. Eng. Med. Biol.*, early access, Feb. 23, 2023, doi: 10.1109/OJEMB.2023.3248307.
[A2] J. Pan, W. Fang, Z. Zhang, B. Chen, Z. Zhang, and S. Wang, “Multimodal emotion recognition based on facial expressions, speech, and EEG,” *IEEE Open J. Eng. Med. Biol.*, early access, Jan. 27, 2023, doi: 10.1109/OJEMB.2023.3240280.
[A3] E. N. Karthik, F. Cheriet, and C. Laporte, “Uncertainty estimation in unsupervised MR-CT synthesis of scoliotic spines,” *IEEE Open J. Eng. Med. Biol.*, early access, Mar. 29, 2023, doi: 10.1109/OJEMB.2023.3262965.
[A4] S. Langarica et al., “Deep learning-based glucose prediction models: A guide for practitioners and a curated dataset for improved diabetes management,” *IEEE Open J. Eng. Med. Biol.*, early access, Feb. 13, 2024, doi: 10.1109/OJEMB.2024.3365290.
[A5] J. Y. Jhang, Y. C. Tsai, T. C. Hsu, C. R. Huang, H. C. Cheng, and B. S. Sheu, “Gastric section correlation network for gastric precancerous lesion diagnosis,” *IEEE Open J. Eng. Med. Biol.*, early access, May 17, 2023, doi: 10.1109/OJEMB.2023.3277219.
[A6] I. E. Toubal, N. Al-Shakarji, D. Cornelison, and K. Palaniappan, “Ensemble deep learning object detection fusion for cell tracking, mitosis, and lineage,” *IEEE Open J. Eng. Med. Biol.*, early access, Jun. 21, 2023, doi: 10.1109/OJEMB.2023.3288470.
[A7] S. Liu, B. Liang, S. Wang, B. Li, L. Pan, and S. H. Wang, “NF-GAT: A node feature-based graph attention network for ASD classification,” *IEEE Open J. Eng. Med. Biol.*, early access, Apr. 26, 2023, doi: 10.1109/OJEMB.2023.3267612.
[A8] Z. Ren, X. Kong, Y. Zhang, and S. Wang, “UKSSL: Underlying knowledge based semi-supervised learning for medical image classification,” *IEEE Open J. Eng. Med. Biol.*, early access, Aug. 15, 2023, doi: 10.1109/OJEMB.2023.3305190.

## References

[1] S. Ghosh, S. Kumar, A. Kumar, and J. Verma, “A closer look at consistency regularization for semi-supervised learning,” in Proc. 7th ACM India Joint Int. Conf. Data Sci. Manage. Data (CODS-COMAD) /11th ACM IKDD CODS Conf. / 29th COMAD Conf., 2024, pp. 10–17.

[2] S. Chen, S. Zhao, and C. Huang, “An automatic Malaria disease diagnosis framework integrating blockchain-enabled cloud–Edge computing and Deep learning,” IEEE Internet Things J., vol. 10, no. 24, pp. 21544–21553, Dec. 2023.

[3] J. Park, J. Kim, and B. Han, “End-to-end learning for weakly supervised video anomaly detection using absorbing Markov Chain,” Comput. Vis. Image Understanding, vol. 236, 2023, Art. no. 103798.

[4] H. Liu, C. Y. Liu, H. R. Zhao, H. Tian, J. W. Liu, and L. J. Tian, “Non-intrusive load monitoring method for multi-energy coupling appliances considering spatio-temporal coupling,” IEEE Trans. Smart Grid, vol. 14, no. 6, pp. 4519–4529, Nov. 2023.

[5] N. Lv, X. Z. Xiang, X. Y. Wang, Y. L. Qiao, and A. El Saddik, “Learning feature contexts by transformer and CNN hybrid deep network for weakly supervised person search,” Comput. Vis. Image Understanding, vol. 239, 2024, Art. no. 103906.

[6] G. Ning, H. Liang, X. Zhang, and H. Liao, “Autonomous robotic ultrasound vascular imaging system with decoupled control strategy for external-vision-free environments,” IEEE Trans. Biomed. Eng., vol. 70, no. 11, pp. 3166–3177, Nov. 2023. 37227912 10.1109/TBME.2023.3279114

[7] J. Kim, Y. K. Kim, H. Kim, H. Jung, S. Koh, and Y. Kim, “Machine learning algorithms predict successful weaning from mechanical ventilation before intubation: Retrospective analysis from the Medical Information Mart for Intensive Care IV database,” JMIR Formative Res., vol. 7, 2023, Art. no. e44763. 10.2196/44763 PMC10685278 37962939

[8] S. Reiss, C. Seibold, A. Freytag, E. Rodner, and R. Stiefelhagen, “Decoupled semantic prototypes enable learning from diverse annotation types for semi-weakly segmentation in expert-driven domains,” in Proc. IEEE/CVF Conf. Comput. Vis. Pattern Recognit., 2023, pp. 15495–15506.

[9] B. Sheng, C. Li, L. Bao, and R. Li, “Probabilistic HIV recency classification-A logistic regression without labeled individual level training data,” Ann. Appl. Statist., vol. 17, pp. 108–129, 2023. 10.1214/22-aoas1618 PMC10577400 37846343

